# The Microbiologically Influenced Corrosion and Protection of Pipelines: A Detailed Review

**DOI:** 10.3390/ma17204996

**Published:** 2024-10-12

**Authors:** Xueqing Lv, Can Wang, Jia Liu, Wolfgang Sand, Ini-Ibehe Nabuk Etim, Yimeng Zhang, Ailing Xu, Jizhou Duan, Ruiyong Zhang

**Affiliations:** 1 School of Environmental and Municipal Engineering, Qingdao University of Technology, 777 Jialingjiang Rd, Qingdao 266000, China; qinglvxue999@163.com (X.L.); xalcsu@sina.com (A.X.); 2 Key Laboratory of Advanced Marine Materials, Key Laboratory of Marine Environmental Corrosion and Bio-Fouling, Institute of Oceanology, Chinese Academy of Sciences, Qingdao 266071, China; wangcan@qdio.ac.cn (C.W.); liujia0013@163.com (J.L.); wolfgang.sand@qdio.ac.cn (W.S.); ininabuk@qdio.ac.cn (I.-I.N.E.); zhangyimeng21314@163.com (Y.Z.); duanjz@qdio.ac.cn (J.D.); 3 Aquatic Biotechnology, University of Duisburg-Essen, 45141 Essen, Germany; 4 Institute of Biosciences, Freiberg University of Mining and Technology, 09599 Freiberg, Germany; 5 Marine Chemistry and Corrosion Research Group, Department of Marine Science, Akwa Ibom State University, Uyo P.M.B. 1167, Nigeria; 6 Guangxi Key Laboratory of Marine Environmental Science, Institute of Marine Corrosion Protection, Guangxi Academy of Sciences, Nanning 530007, China

**Keywords:** microbiologically influenced corrosion, pipeline corrosion, metallic materials, biofilm

## Abstract

Microbial corrosion is the deterioration of materials associated with microorganisms in environments, especially in oil- and gas-dominated sectors. It has been widely reported to cause great losses to industrial facilities such as drainage systems, sewage structures, food-processing equipment, and oil and gas facilities. Generally, bacteria, viruses, and other microorganisms are the most important microorganisms associated with microbial corrosion. The destructive nature of these microorganisms differs based on the kind of bacteria involved in the corrosion mechanism. Amongst the microorganisms related to microbial corrosion, sulfate-reducing bacteria (SRB) is reported to be the most common harmful bacteria. The detailed mechanistic explanations relating to the corrosion of pipelines by sulfate-reducing bacteria are discussed. The mechanism of microbial corrosion in pipelines showing the formation of pitting corrosion and cathodic depolarization is also reported. The current review provides theoretical information for the control and protection of pipelines caused by microbial corrosion and how new eco-friendly protection methods could be explored.

## 1. Introduction

**Figure 1:**
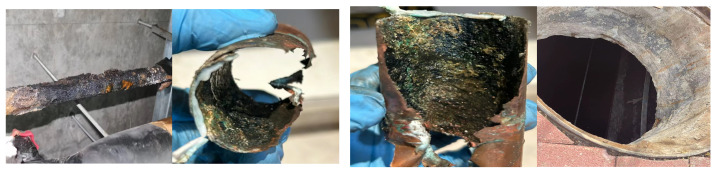
Pipeline corrosion of marine and residential facilities in Qingdao, China.

Globally, pipelines have been used in many industrial applications to transport large volumes of petroleum products, crude, and natural gas within a desired location. For instance, the use of long-distance oil and gas pipelines in China is growing at a rate of more than 5000 km per year and is expected to exceed 240,000 km by 2025 [1]. Applying these pipelines in several environments could expose them to harsh natural environments such as the marine (deep sea) or underground soil environments. Another significant issue faced by pipeline materials is the deterioration due to the influence of corrosion actions, as shown in Figure 1 [2]. The data from a national corrosion survey show that the total corrosion cost in China in 2014 was about RMB 2 billion, accounting for 3.3% of the gross national product [3,4]. Among the existing types of corrosion that cause environmental issues, microbiologically influenced corrosion (MIC) is one of the main causes of pipeline corrosion. MIC accounts for about 20% of the total cost of the corrosion of metallic materials [4,5,6].

MIC can occur almost everywhere in seawater, fresh water, soil, the air, and in a variety of technical environments such as drinking-water systems, food-processing facilities, medical instruments, oil transportation infrastructure, and drainage pipes (Figure 1) [7]. MIC has various characteristic modes, such as accelerated pore corrosion, crevice corrosion, hydrogen embrittlement, subsurface corrosion, and selective dealloying corrosion, etc., which shorten the service life of alloys and other materials and pose a serious threat to important facilities such as those employed in wind power, aviation, shipping, nuclear power, etc. [8]. Therefore, investigations on the corrosion and protection mechanisms of pipelines and a comprehensive analysis of the role of microorganisms in the corrosion process are advantageous for reducing the cost of pipeline corrosion and also for the prevention of the occurrence of accidents.

MIC is an electrochemical process that causes or accelerates the corrosion of metallic or non-metallic materials in the presence of microorganisms [9]. MIC does not alter the basic electrochemical principle of corrosion but rather accelerates the corrosion process by promoting anodic dissolution and cathodic depolarization [10,11]. Numerous species of microorganisms are involved in MIC, and these include bacteria, archaea, fungi, etc. [9,12,13]. MIC can be divided into two categories, namely, aerobic MIC and anaerobic MIC [14]. The common microorganisms involved in aerobic MIC processes are sulfur-oxidizing bacteria (SOB) [15,16] and iron-oxidizing bacteria (IOB) [17]. They consume oxygen and produce acidic substances which enhance metal oxidation, leading to corrosion. For anaerobic MIC, sulfate-reducing bacteria (SRB) [18,19] are the notable anaerobic corrosion-causing microorganisms.

**Figure 2:**
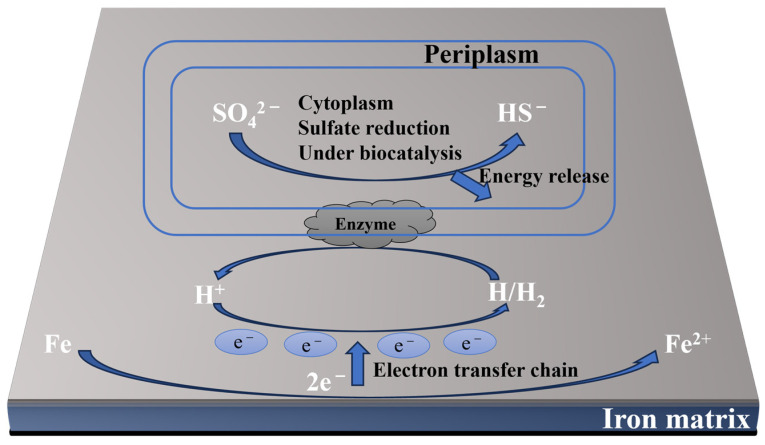
The mechanism of sulfate reduction catalyzed by biological cathodes.

Only a few sulfates and other reduced sulfur compounds act as an electron acceptor for growth [20]. As shown in Figure 2, SRB directly obtains electrons from the metal surface [21,22]. Consequently, the biofilms on the metal surface enhance the oxidation process leading to pit corrosion. Many studies have shown that the attachment of microorganisms leading to biofilm formation could depend on the nature of pipeline materials [23,24].

Currently, the primary pipeline materials are concrete, copper, galvanized steel, cast iron, stainless steel, polypropylene (PP), and polyvinyl chloride (PVC). The different materials have different applications and corrosion resistance (Table 1) [21,25]. Concrete is the most widely utilized pipeline material, and the corrosion problem is particularly severe in concrete pipelines used for sewage transport. This is due to the complexity of the internal environmental conditions, the high concentration of substrates, and an anaerobic–aerobic alternating internal environment [26]. Wang [27] et al. conducted a review on the mechanisms of microbial influence on concrete corrosion in sewer systems. Their review emphasized that acidophilic microorganisms play a significant role in accelerating the deterioration of concrete pipelines. According to Ji et al. [28], the rate of biofilm development on different materials showed that copper pipes may inhibit an effectively short-term biofilm formation in comparison to concrete. Furthermore, copper demonstrates an effective inhibition of bacterial growth, the maintenance of water quality, and good corrosion resistance against chemical substances and oxidants in water [29,30]. PP pipes are currently the most utilized water pipe in construction due to their unique advantages: non-toxic, lightweight, corrosion resistance, and pressure resistance [31,32,33]. Plastic pipes have a smooth surface, are durable, and have superior corrosion resistance compared to metal pipes. Weronika et al. [34] reported that plastic PVC and polyethylene (PE) pipelines are likely susceptible to pathogenic bacteria compared to metal pipes. Furthermore, plastic pipelines are susceptible to deformation and thermal decomposition under high temperatures, resulting in the loss of function and the generation of microplastic particles, attracting strong attention as hazardous substances. It has been estimated that on average, humans may ingest about one “credit card” account of microplastic particles per week [35]. Consequently, there is an urgent need for research aimed at mitigating the corrosion of pipeline materials and minimizing environmental contamination from corrosive byproducts.

The current research into pipeline protection against MIC follows similar approaches to conventional corrosion techniques. The newly developed protective coatings and corrosion inhibitors have reduced corrosion rates in many industrial applications. However, the complexity of the microbial community and environment where pipeline materials are used makes it difficult to mitigate MIC. Some other factors limiting the mitigation of MIC include microbial interspecies relationships, materials, and their microstructure. These factors are rarely measured, which makes it difficult to comprehensively and adequately evaluate the problem caused by MIC.

## 2. Types and Principles of Pipeline Corrosion

### 2.1. Carbon Steel

Carbon steel is a commonly used raw material in industrial applications due to its high strength and cost-effectiveness [61]. Its carbon content ranges between 0.0218% and 2.11%. The range is divided into low-, medium-, and high-carbon steel. The higher the carbon content, the greater the hardness and strength but the lower the plasticity and toughness [62,63]. In pipeline systems, the steels utilized are medium- and low-carbon steel. Li et al. [64] proved that under identical conditions, SRB was likely to form a dense biofilm on the surface of carbon steel, making the material susceptible to corrosion by SRB. Furthermore, the density of attached cells on the surface of a carbon-steel biofilm is two orders of magnitude higher than that of a stainless-steel biofilm. These findings indicate that carbon steel is more susceptible to corrosion.

### 2.2. Stainless Steel

Stainless-steel pipes are used in many fields due to their excellent corrosion resistance, high-temperature resistance, and mechanical strength. According to the material, these pipes are divided into austenitic, martensitic, and ferritic stainless-steel pipes, etc. [65]. The two common ones on the market are 304 and 316 [66]. Stainless-steel pipes can be used for indoor and outdoor piping systems, such as water supplies, drainage, and heating pipes, because stainless steel, in the presence of sufficient oxygen, can easily form a passivation film that protects from corrosion [67,68]. A study by Sun et al. [69] showed that dissolved oxygen is a key factor for the ammonia corrosion of stainless steel. It was reported that the corrosion resistance of stainless steel was little affected under low- or high-concentration ammonia, if sufficient dissolved oxygen was available. It causes the passivation and corrosion resistance of stainless steel. The presence of some disinfectants can also increase the corrosion of stainless steel [70,71].

### 2.3. Copper

Copper and copper-based alloys are reported to have poor corrosion resistance compared with steel [39,72]. It has been reported that scales are easily formed on copper pipelines [73]. There are three types of scales: the first is the compound that crystallizes directly from the flowing water on the surface of a pipe (mostly contains calcium carbonate or magnesium and is distributed evenly on the inner surface of a pipe); the second is composed of scale deposits transported by flowing water from other sites; the third is the scale formed by corrosion products (characteristic of unlined cast iron pipes and copper pipes). Furthermore, copper is a non-passive metal, where oxygen corrosion occurs. Under the condition of sufficient oxygen, the corrosion rate is high. Under hypoxia, the corrosion rate decreases and the resistance increases, due to a decrease in the current and fluctuation of cathodic depolarization. This inhibits the anodic reaction of copper in an environment with ammonia. The main factors affecting copper corrosion include pH levels, temperature, total organic carbon, dissolved inorganic carbon, and chloride ions [74,75,76,77,78,79].

### 2.4. Polymeric Organic Materials

Polymeric organic materials are important in industrial applications. These materials are resistant to acidic or alkaline media and can be adopted for the construction of buildings [80]. Polymeric materials are not easily destroyed by scale formation. Corrosion is mainly caused by the formation of a biofilm [23] and the change of its mechanical characteristics caused by the degradation or loss of various chemical components. The reaction between water and polymeric materials can cause changes in water quality, especially for pipes. Studies have shown [81,82] that water-quality parameters (such as sulfates, bicarbonate and humic acids, or pH levels) also affect the release of metallic elements from the inner surface. It is important to note that the velocity at which water flows determines how quickly nutrients are absorbed by the water, thus promoting the growth of biofilms. Moreover, due to the composition of polymeric pipes, biofilms are formed. On the other hand, the use of polymeric pipes has reduced problems associated with heavy metals. The pressure resistance of these pipes is poor. They are not suitable for high-pressure applications.

## 3. Mechanism of MIC of Pipelines

MIC is considered to be caused by the presence and activity of various microorganisms [83]. From a microbial perspective, the attachment of microorganisms to metallic materials indicates their lifestyle. Microorganisms use metallic materials to attach and possibly as sources of energy [9].

On the one hand, the metabolites of microorganisms can have corrosive effects on materials. Microorganisms produce a variety of organic and inorganic substances, such as acids, sulfide, ammonia, etc. These substances are highly corrosive and can react chemically with metal, resulting in corrosion. On the other hand, the adhesion and aggregation of microorganisms can have corrosive effects on the material. Microorganisms attach to the surface of materials and form microbial communities. These extracellular polymeric substances (EPSs) can cause physical and/or chemical damages.

Corrosion in pipeline systems is mainly caused by sulfate reduction, methanogenesis, and fermentation processes. The microorganisms at the top of a pipeline are usually composed of some aerobic bacteria, while corrosion at the bottom of a pipeline is mostly caused by anaerobic bacteria [26]. One reason for this is that at the bottom of a pipeline, an anaerobic environment exists, which is conducive to the growth of anaerobic bacteria. Another reason is that the aerobic bacteria at the top of a pipeline use oxygen. This is more conducive to the growth and reproduction of SRB, thus accelerating corrosion.

### 3.1. Sulfate-Reducing Bacteria on Concrete Corrosion

**Figure 3:**
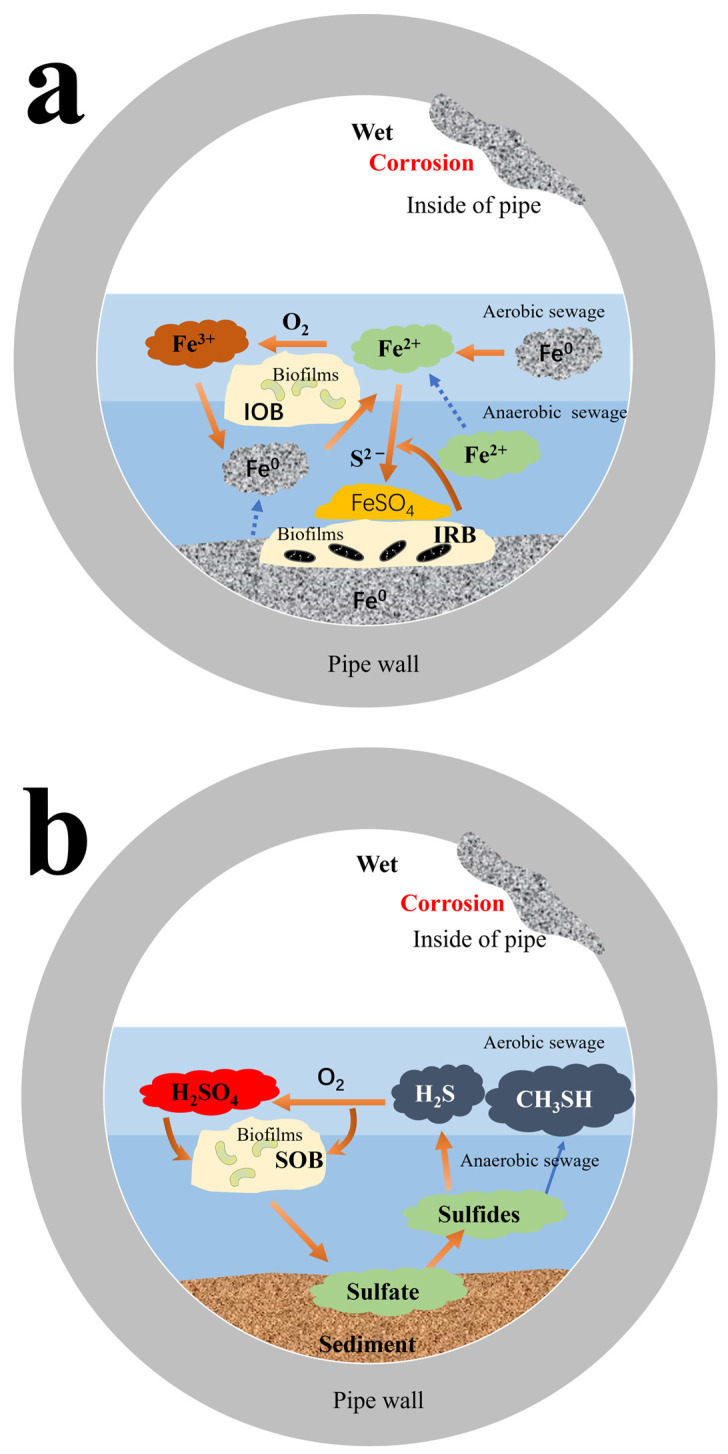
The corrosion caused by microorganisms in concrete piping systems is mainly involved in the iron cycle (**a**) and sulfur cycle (**b**) process [27].

Sewage contains sulfate and various organic compounds. A sewage environment is known to create an anaerobic environment conducive to the growth of SRB. SRB converts sulfate into hydrogen sulfide gas and CH_3_SH. In the turbulent action of sewage, hydrogen sulfide gas (H_2_S) is discharged into the air, and when this H_2_S reaches a certain concentration, it is reduced by oxygen to sulfur (S). Under aerobic conditions, SOB oxidizes sulfur to sulfuric acid (H_2_SO_4_) and corrodes pipes. The sulfate can be transferred to the sewage, forming a sulfur cycle process (Figure 3b).

In concrete piping systems, reinforced concrete is one of the most common materials. Its main component is carbon steel (CS). CS contains iron. A schematic diagram of the iron cycle in a concrete pipeline is shown in Figure 3a. Like the sulfur cycle, the bottom of a pipe is an anaerobic environment, where a large number of corrosive bacteria and ferrous substances appear. Under aerobic conditions, Fe^2+^ is oxidized to Fe^3+^ by IOB with deposition formulation. Iron-reducing bacteria (IRB) can accelerate the cycling process of Fe^3+^ coupled with Fe^0^, producing Fe^2+^.

### 3.2. Sulfate-Reducing Bacteria on Steel Corrosion

#### 3.2.1. Carbon Steel Corrosion

**Figure 4:**
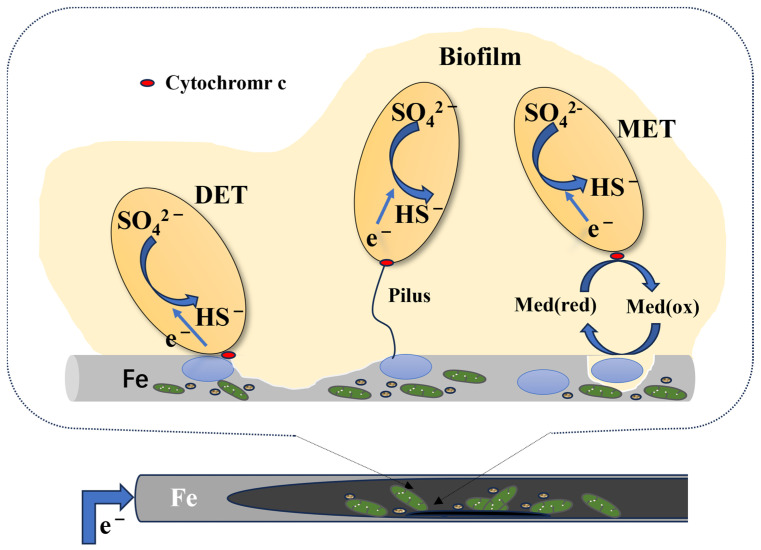
Modes of direct electron transfer during SRB corrosion of steel.

Carbon steel (CS) is often prone to corrosion when exposed to a microbial environment [64]. For example, SRB can form a dense biofilm on the surface of carbon steel, leading to the deterioration of the material. SRB has emerged as the most extensively researched anaerobic microorganism for pipeline corrosion due to its characteristic features [84]. According to some research, pitting corrosion, due to the presence of SRB, is more severe than uniform corrosion. The concentration of ferrous ions in an SRB medium has effects on hydrogen sulfide generation, the number of floating cells, and the pH of the media. This indicates that the initial [Fe^2+^] plays a crucial role in the SRB MIC of carbon steel and promotes corrosion rates and pitting corrosion (based on the theory of biocatalytic cathode sulfate reduction, BCSR) [85] (Figure 4). Zero-valent Fe is, for many types of SRB, an electron donor (Equation (1)). The electrons released from zero-valent Fe pass through the SRB cell wall and enter the cytoplasm via the EET–MIC pathway [86,87]. The sulfate-reduction reaction then takes place with sulfate as the electron acceptor (Equation (2)). The whole corrosion process is based on corrosion kinetics and thermodynamics.

(1) $$\mathrm{Oxidation}:\;\mathrm{Fe}\to Fe^{2+}+2e^{-}\;(in\;vitro)$$

(2) $$Reduction:S{O_{4}}^{2-}+9H^{+}+8e^{-}\to HS^{-}+4H_{2}O\;(in\;vivo)$$

**Figure 5:**
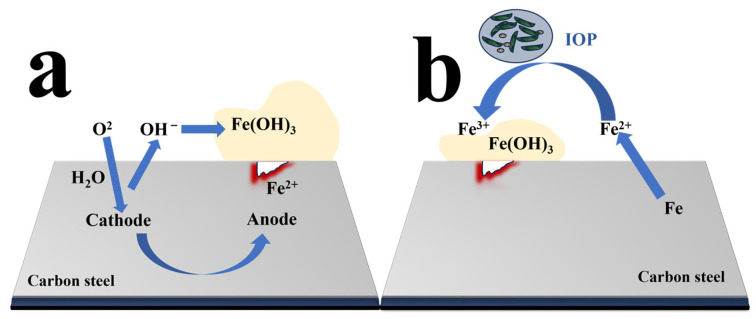
Iron reaction pathways of pitting potentially caused by oxygen and Fe(OH)_3_, precipitation (**a**), and crevice corrosion in the presence of IOB (**b**).

The main form of corrosion affecting carbon steel is pitting corrosion caused by SRB. The pitting mechanism is shown in Figure 5. MIC pitting is connected with the bacterial distribution, biofilm, and ion selectivity of corrosion products [88,89]. Some ions are noted to flow through the biofilm matrix. In this case, the buildup of H^+^ ions within the substrate surface, underneath the biofilm, may result from this ion selectivity. Furthermore, a localized increase in the H^+^ concentration can produce an acidic environment, dramatically reducing pH levels. The anodic deterioration of the substrate accelerates through the accumulation of H^+^ ions close to its surface. In most cases, the pitting corrosion rate may be accelerated by cations reacting with other anions in the environment to create soluble complexes [90].

**Figure 6:**
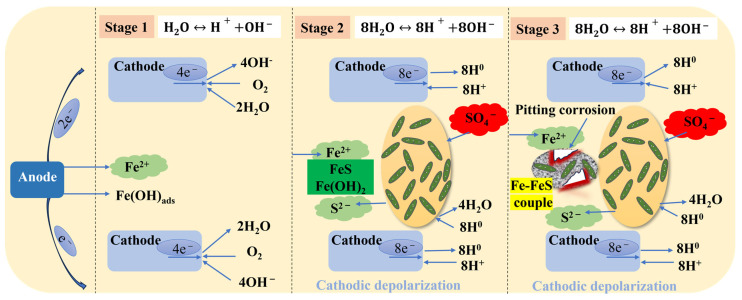
Schematic diagram of the electrochemical process of SRB corrosion at cracks.

Figure 6 shows a schematic of the electrochemical process of SRB corrosion. The factors influencing of the corrosion process include the potential difference, pH levels, stress levels, etc. [91]. The stress level is divided into yield stress and plastic stress. Without cathodic protection, the higher the stress level is, the more severe the pitting corrosion is [92].

#### 3.2.2. Stainless Steel Corrosion

In contrast to CS, the surface of stainless steel (SS) forms a thin and stable passivation film, which causes excellent corrosion resistance [93].

Consequently, the degree of MIC pitting attacks on stainless steel is known to be lower than that of carbon steel. The pitting corrosion of both types of steel is more serious under anaerobic than aerobic conditions. Ru [94] et al. studied the anaerobic corrosion of 304 stainless steel by *Pseudomonas aeruginosa* under strict anaerobic conditions. The rate of pitting corrosion after day 14 was higher compared to day 7. Also, the pH of the solution in the pitting process was alkaline. It was concluded that the corrosion of 304 stainless steel by *Pseudomonas aeruginosa* was not caused by organic acids and other excreted corrosion products but by the release of electrons through iron oxidation. Zhang et al. [95] studied the corrosion effect of SRB on stainless steel and found that stainless steel corroded more under anaerobic conditions than aerobic conditions. In addition, the pitting degree of SRB on stainless steel under strict anaerobic conditions was higher than that of *Pseudomonas aeruginosa*.

### 3.3. Nitrate-Reducing Bacteria on Copper Corrosion

Nitrate-reducing bacteria (NRB) are the primary bacteria responsible for MIC in copper pipelines. Studies have shown the effect of nitrate-reducing bacteria on copper corrosion [91,92]. Xu et al. described MIC activities on copper steel. Their study found that the corrosion process was initiated by EET–MIC, causing the formation of Cu_2_O and Cu(NH_3_)_2_ [96]. The sessile cells on the surface of copper produce NH_3_, while Cu reacts with NH_3_ to form Cu(NH_3_)_2_, which is converted into the final corrosion product (Cu_2_O). In addition, the bacteria reduce the DO content, while NO_3_^−^ and DO jointly decrease the anodic current density, resulting in a change in the surface state of Cu, leading to corrosion.

The biofilm of *P. aeruginosa* can be converted into a biomineralizing film which protects the metal from corroding. However, if the concentration of nitrate increases, the formation of the biofilm becomes less stable, increasing the total number of bacteria. The uniform corrosion of copper pipes can be accelerated, resulting in severe corrosion. Additionally, nutrients in the environment affect biofilm formation, like the ratio of nutrients in the medium, such as organic C, inorganic N, and P [72].

### 3.4. Plastic Pipes Corrosion

There are numerous types of plastic pipes, with PVC serving as a representative material. PVC pipes are among the most prevalent types of plastic pipes. PVC consists of polyvinyl chloride as its principal component. The primary microorganisms causing damage to PVC pipes are small filamentous fungi. The presence of bacteria and molds can alter the plastic-related properties of the material. The decomposition of polymer substances and the production of microbial metabolites can also contribute to the deterioration of PVC. It is, therefore, crucial to incorporate antifungal and antibacterial agents into the production of the raw materials of PVC pipelines [97].

For industrial applications, plastic pipes are subject to a variety of corrosion processes, including but not limited to chemical corrosion, wear corrosion, and microbial corrosion. Chemical corrosion represents the most prevalent form of corrosion. It is attributed to some factors, including ozone compounds, organic solvents, temperature, pH values, and other variables within the medium. Wear corrosion refers to the corrosion phenomenon caused by the friction of solid particles or fluid media within the pipeline. This phenomenon is most prevalent in the relatively narrow sections of plastic pipes. In the case of plastic pipes, the primary factors influencing their corrosion are the composition of the material and the medium through which they are conveyed. Research indicates that utilizing PVC pipes may reduce costs and may also mitigate metal corrosion and rust in humid areas of fish-farming facilities [98]. As a vital conduit for the transportation of fluids, a PVC pipe can be subjected to appropriate protective measures during its production to extend its service life and to ensure the safety of the manufacturing process.

## 4. Factors Affecting Corrosion of Pipeline MIC

To identify effective methods for the prevention and control of pipeline MIC, it is essential to conduct a comprehensive study of the composition of the microbial community. A sewage environment hosts many microorganisms, including bacteria, archaea, and fungi. These microorganisms exert a profound influence on the corrosion of pipelines, while also playing a pivotal role in maintaining the equilibrium of the surrounding environment within the pipeline. The metabolic activities of microorganisms are influenced by factors such as environmental factors and the pipeline materials of biofilms. To identify methods for mitigating MIC in pipelines, it is essential to investigate the influencing factors of MIC in pipelines.

### 4.1. Environmental Factors

#### 4.1.1. Temperature

Temperature is one of the factors required for the manufacturing of different pipes. As living standards have improved, adopting hot-water systems has become increasingly prevalent. It is also noteworthy that sewer systems are becoming increasingly heated. Concurrently, the temperature provides an optimal environment for bacterial growth, thereby accelerating the corrosion of the pipeline caused by microorganisms. Sand et al. [99] reported that the average temperature increase in a sewer system is about 7 °C. This also has higher requirements for the temperature resistance of pipeline materials. Pipeline systems vary in their temperature-resistance requirements. For instance, hot- and cold-water systems necessitate high-temperature-resistant materials to ensure the safe transmission of hot and cold water. Conversely, conventional sewage or water supply pipelines do not cause the imposition of mandatory requirements on the heat resistance of pipes. Based on Jason et al.’s [100] study, the temperature gradient of a residential drinking-water system was simulated by changing the direction of the pipeline (horizontal or vertical). The surface and water temperature of the pipeline were monitored at various intervals to determine the impact of different temperature gradients on copper release. Furthermore, the accelerated thermal current resulted in corrosion during the heating process, thereby accelerating the corrosion rate of copper. Zhao et al. [101] conducted a simulation of high-temperature conditions in deep oil wells and studied the temperature and corrosion resistance of anode materials. Their findings indicate that a sacrificial anode can significantly reduce the corrosion rate of microbial pipelines under high-temperature conditions.

#### 4.1.2. pH

Several studies have demonstrated that pH affects the composition, abundance, and corrosion of bacterial communities in MIC [41,99,102,103]. This is because the pH value of a concrete surface continues to decline with time, eventually becoming acidic [104]. Some sulfur-oxidizing bacteria and iron-oxidizing bacteria (e.g., *Acidic thiobacillus*, *Thiobacillus ferrooxidans*, *Bacillus thiooxides*, etc.) can participate in the sulfur and iron cycles during the corrosion process. Jiang et al. [105] inoculated wastewater on the surface of concrete samples through a simulated sewer system. Their findings indicate that SOB accounted for 80–90% of the microbial community, with *A. thiooxidans* accounting for 35–50%. Greng et al.’s [103] study identified sulfur-oxidizing bacteria as the dominant strains responsible for the severe corrosion of sewer systems. In summary, microbial metabolites result in a reduction in the pH value of a groundwater environment, which in turn leads to the degradation of concrete pipelines [91]. This, in turn, is conducive to the growth of corrosive microorganisms, resulting in severe corrosion. Zhou et al. [102] conducted research on concrete corrosion and related microbial communities by varying the salt concentration of the concrete pipes of a marine sewage system. Their findings reveal that corroding microorganisms can decompose macromolecular organic matter, providing energy for their growth and reproduction and promoting bacteria growth. This process results in the deterioration of concrete, and its structural properties, leading to a high corrosion rate.

#### 4.1.3. MPs

Plastics can be classified into two categories based on their size: macro-plastics (diameter > 5 mm) and microplastics (diameter < 5 mm) [106,107]. The question regarding plastic or metal pipes forming biofilms on their pipe walls is of immense interest. Such biofilms can retain and accumulate microplastics and compounds in the water. When the flow conditions or water parameters are altered, the biofilm will detach from the pipe wall, and the microorganisms within the biofilm will accelerate the corrosion of the metal pipeline. This applies, in particular, to heavy metals and microplastics, which will increase exponentially, leading to a deterioration in both the quality of pipelines and water quality.

The sources of microplastics include wastewater, oceans, agriculture, and other environments. These microplastics enter the human body from different pathways, causing significant harm to human health. Microplastics can alter the structure, composition and functional characteristics of bacteria. Given that MPs can transport specific bacterial groups and that microorganisms play an important role in pipeline corrosion, it can be reasonably concluded that microplastics are also closely related to pipeline corrosion. The bacterial community that parasitically inhabits the surface of aquatic microplastics (MPs) exhibits notable differences from the surrounding environment [108]. MPs can serve as carriers for the transportation of microorganisms and pollutants. In specific environments, factors such as a prolonged residence time in the pipeline, shallow depth, high ultraviolet penetration, low water pressure, and high temperature can influence the selection of particular microflora aggregation in plastics [108]. Furthermore, microplastics can serve as a protective barrier for microorganisms, enhancing their survival rate in the face of environmental stressors. Harmful bacteria may colonize the surface of MPs. Several studies have indicated that MPs may pose a risk to human health [109].

### 4.2. Biofilms

Biofilm formation not only accelerates the corrosion of microorganisms on metal pipes but also reduces the stability of non-metal pipes and even provides favorable growth conditions for pathogens [110]. The total number of bacteria on the biofilm of a pipe is significantly greater than that in water [111]. The bacteria in the biofilm on a pipeline are predominantly coccus and bacillus, but the distribution of the microbial community structure on different pipes is not uniform. Due to its inert material, a PPR pipe is not conducive to the growth of dominant heterotrophic bacteria, even though the population diversity is high. The population of dominant heterotrophic bacteria is not dominant on a PPR pipe. The population distribution of copper pipes is relatively simple due to metal ions, while the dominant bacterial community of galvanized-steel pipes is situated between the two. Furthermore, opportunistic pathogens do not constitute a significant portion of the dominant heterotrophic bacteria in PPR pipes or the epiphytic biofilm on copper pipes. From the perspective of drinking-water safety, copper pipes are a more suitable choice for water supply networks, and the corrosion problem of copper pipes and other metal pipes requires further investigation [112].

### 4.3. Pipeline Materials

Pipeline materials are important for the flow of gases and liquids in different industries. Various pipeline materials are chosen based on their design and operating factors, intended applications, and operational requirements [113,114]. Different pipeline materials will have different effects on the formation of biofilms in the pipeline system [111], the diversity of microbial populations on a biofilm [112], and the distribution of epibiotic biofilm microorganisms [110]. It is important to note that the grade of pipeline materials could also influence biofilm formation, depending on the environment [115,116].

The industrial and international best practices from the American Petroleum Institute (API), American Society of Mechanical Engineers (ASTM), and American National Standards Institute (ANSI) provide detailed specifications for selecting pipeline materials [115].

## 5. Monitoring and Protection Technology of Pipeline MIC

### 5.1. Monitoring

The most common methods employed for the protection of pipelines from microbial corrosion include physical, chemical, biological, coating, and other methods. However, the complexity of the buried pipeline environment renders physical and chemical methods challenging to implement in practice. Currently, research on MIC protection mechanisms is primarily focused on corrosion mechanisms and the development of antibacterial materials. In the context of research into the corrosion-protection performance of buried pipelines and soil microbial monitoring and detection technology plays a pivotal role in the investigation of microbial corrosion-protection mechanisms. The monitoring of soil microbial diversity facilitates the elucidation of the law governing changes in soil microbial composition and diversity, the establishment of a relationship between changes in soil microbial diversity and the corrosion rate of buried pipeline materials, and the provision of a robust foundation for the explanation of the microbial corrosion process of buried pipelines [117].

### 5.2. Protection

#### 5.2.1. Coating

Coating serves to prevent the adhesion of microorganisms by improving the smoothness of the metal surface. For instance, a novel iron-based amorphous coating was developed through thermal spraying to reduce the thickness of a biofilm and the number of living cells on the surface. Zheng et al. [118] conducted a study to analyze the corrosion behavior of micro-arc (MAO) coatings of 80 μm and 30 μm on the surface of AA2024. Their results indicate that the MAO coating of 30 μm exhibited a superior protective effect on AA2024, as evidenced by the observation of the surface of the MAO film and the SRB biofilm. In concrete pipeline systems, a common engineering practice is to coat the pipeline surface with a protective layer. Concrete protective coatings are typically classified into two categories: inert coatings and antibacterial coatings. The primary function of the former is to serve as a barrier, preventing the penetration of biological sulfuric acid into the concrete interior. The latter’s mechanism of action involves reducing the production of biological sulfuric acid by inhibiting microbial activity and even microbial inactivation, thereby safeguarding concrete from such corrosive agents [119,120]. In a study by Parra et al. [121], a single layer of graphene and boron-hydrogen nitride material was coated onto a copper substrate. The interaction between the bacteria and the coated copper surface was then analyzed. Their results indicate that the copper surface treated with graphene changed from a hydrophilic to a hydrophobic surface. As the hydrophobic surface of the material increased, its antibacterial adhesion ability significantly enhanced, effectively reducing the corrosion of copper by bacteria.

Although new antibacterial steel pipelines and antibacterial coatings have demonstrated efficacy in preventing microbial corrosion, significant challenges remain in their development and application. Adversely, there is a lack of clear theoretical understanding of the mechanism of antibacterial action on the formation and corrosion of bacterial biofilms. Since the coating method is a commonly used pipeline anti-corrosion method, further research is required to address several key issues. These include the adhesion between the coating and the pipe wall, the influence of the coating on the pipe properties, and others.

#### 5.2.2. Bactericide and Corrosion Inhibitors

Chemical methods, like fungicides and corrosion inhibitors, are employed to oxidize active enzymes. In oil and gas pipelines, a range of fungicides are employed. However, using fungicides may not only have adverse effects on the water microenvironment but may also induce microbial variation. Furthermore, the long-term use of these fungicides may result in the development of drug-resistant bacteria, which will consequently diminish their anti-corrosion efficacy. Fungicides can be classified into two categories: oxidizing and non-oxidizing. The corrosion action of microorganisms on metal pipes can be significantly inhibited by reducing the activity of microorganisms. Consequently, research into fungicides has become a prominent area of focus. Fungicides can impede the metabolic processes, reproduction, and even the destruction of microorganisms, reducing the production of biological sulfuric acid. Fungicides play a pivotal role in the inhibition of microbial pipeline corrosion.

Green fungicides are non-toxic, abundant, inexpensive, and environmentally friendly [122]. Narenkumar et al. [123] used ginger extract (GIE) as a green fungicide to control biofilm formation and mass spectrum corrosion. Their results show that GIE inhibited biofilm formation by changing its phenotypic characteristics. The water extract of GIE inhibited the growth of bacteria on the surface of MS, and the corrosion inhibition rate of MS reached 80%. Xu et al. [124] indicated that the key to MIC control was the slow release of biofilms. They speculated that tyrosine and some other D-amino acids might be signals of biofilm diffusion. Through experiments, D-tyrosine was found to be an effective enhancer of the green fungicide tetramethyl sulfate (THPS). It was found that when D-tyrosine combined with THPS, using the synergistic effect between its two chemical substances can not only inhibit the formation of an SRB biofilm on the surface of C1018 mild steel but can also greatly reduce the dose of THPS, which can reduce environmental and water pollution. Although the bactericidal effect and corrosion-inhibition performance of the above two fungicides are good, if the same fungicide is used for a long time, it will not only increase the resistance of microorganisms but will also pollute the water environment and will eventually lead to a decline in the bactericidal effect.

#### 5.2.3. Photocatalysis and Electrocatalysis

In the oil and gas industry, cathodic protection is widely used in the anti-corrosion technology of steel pipes. This is a cathodic anti-corrosion technology that turns the whole pipe into a corroding battery [125]. In recent years, the use of N-type semiconductors for the photoelectrochemical cathodic protection of metal materials has been given more and more attention as an important anti-corrosion technology. This electrochemical protection method has been widely used for metal corrosion protection in marine buildings, ships, underground steel, and other industries, including groundwater pipeline systems.

The mechanism of inhibiting the MIC of stainless steel involves the biofilm consuming dissolved oxygen on the metal surface through aerobic respiration and reducing the oxygen concentration in contact with the metal surface. The biofilm can prevent the diffusion of O_2_ to the metal surface, can slow down the reaction of bacteria with the metal surface, and can inhibit MIC [126,127,128]. An increase in the dissolved oxygen (DO) content can inhibit the growth of SRB, and SRB accumulates to form a local anaerobic environment suitable for growth and metabolism for its own survival needs. Some studies have found that an increase in the DO content will lead to slow SRB metabolism, so that the SS passivation film can be better repaired, thus reducing the corrosion rate of SS by microorganisms under aerobic conditions. In recent years, it has been found that some electroactive biofilms can also transfer electrons to the surface of SS pipelines by oxidizing organic electron donors in the media, which can inhibit the pitting corrosion of SS pipelines [129,130]. Electroactive biofilms (EABs) [131,132] are microbial membranes formed on the surface of conductive solid materials and are capable of exchanging electrons with the substrate without an external mediator.

The principle of photocatalytic antifouling is a water treatment method that uses light to stimulate photosensitive semiconductor materials to produce more electrons and holes to accelerate the oxidation and reduction reaction so that toxic pollutants can be degraded into less- or non-toxic substances. An advantage of this method is that it can utilize inexhaustible solar energy as well as semiconductor materials to achieve the conversion of light energy into chemical energy through an energy level transition to produce more bactericidal substances and to protect the pipeline from corrosion, instead of the traditional cathodic protection law that sacrifices the anode. Laoun et al. [125] used the impressed current of solar photovoltaic panels to optimize the protective effect of the combination of solar energy and cathode elements on a pipeline, thus achieving the cathodic protection standard and ensuring the safe transmission of the pipeline. The principle purpose of electrocatalysis antifouling is to promote or inhibit the electron transfer reaction on electrodes by changing the current or other electrochemical parameters, that is, to change the specificity of the material by changing its composition so as to achieve more ways to degrade or kill bacteria, such as hydrogen peroxide and other substances with strong oxidation, which can degrade or even kill harmful bacteria.

With the development of photocatalysis and electrocatalysis, photocatalysis has become an environmentally friendly metal-conservation technology. Liu et al. [133] prepared N-TiO_2_ derived from a metal–organic framework (MOF) by the NH_2_-MIL-125 precursor system for the photocatalytic degradation of methylene blue and found that N-TiO_2_ has high degradation efficiency. N-TiO_2_ generates photoelectron–hole pairs under photoelectric action, which promotes the electrocatalytic two-electron water oxidation reaction (2e-WOR), thus accelerating the photogenerated holes to oxidize water to hydrogen peroxide and hydrogen peroxide to degrade methylene blue.

#### 5.2.4. Physical, Modeling, and Biological Methods

Physical methods, like ultraviolets; improving ultrasound function by destroying the SRB biofilm, thereby killing the SRB; and installing ultraviolet sterilization devices in the pipeline delivery system can mitigate these effects. However, the environment of the buried pipeline can be difficult to predict, thus limiting the application of physical methods.

In a concrete pipeline system, corroded concrete is usually observed by scanning electron microscopy (SEM), and the corroded concrete is typically analyzed by an energy dispersive spectrometer (EDS) and X-ray fluorescence. In addition to physicochemical and biological analysis methods, some researchers use modeling methods to measure the degree of concrete deterioration [134,135,136]. The pH level and hydrogen sulfide and oxygen concentrations in different thicknesses within the biofilm were measured by microsensors [137]. All chemical parameters were found to impact an in-depth study of microbial concrete corrosion [138]. Signature lipid biomarker fatty acids of polar lipids (PLFAs) can be used to define the microbial biomass and colony structure in biofilms, soils, and sediments. Kerger et al. [139] pointed out that the specificity of the PLFA model could determine the presence of *Thiobacilus acidogenes* and could thus better identify bacterial colonies in corroding concrete pipes. However, there is no uniform standard to measure the degree of deterioration of concrete in the world, so we need a unified system to characterize the corrosion of concrete pipes.

The biological method refers to the interspecific relationship of microorganisms (such as competition or antagonism) to inhibit the growth of corrosive microorganisms. With the continuous innovation of anti-corrosion means, to reduce the corrosion degree of pipelines, a new anti-corrosion means was proposed, which is to reduce the abundance of bacteria causing pipeline corrosion by using the interspecific relationship of organisms. *Pseudomonas aeruginosa* is famous for its strong corrosion. Liu et al. [140] found that *Thiobacilus denitriformis* could oxidize the reducing sulfide produced by SRB to SO_4_^2−^, thereby reducing the corrosion of SRB metabolites. Although the cost of this method is low, the actual environment is difficult to measure, and the amount of microorganisms added is difficult to control, which is easy to cause a large number of microorganisms to grow and multiply, produce pathogenic bacteria, and pollute water quality to the extent of endangering human health.

## 6. The Reliability of the Corrosion Protection Method

In corrosion studies, the reliability of the corrosion protection method is important. Some corrosion protection methods stated in this review have shown enormous industrial applications, but their reliability depends on some factors like the environment, pH levels, and materials. Among them, the physical method has been eliminated by most anti-corrosion options due to its limitations in applications. The biological technique is difficult to control because of the various uncertain factors of strains. Some could produce pathogenic bacteria, so there is a great risk to the human body or the environment. In the long-term use of fungicides, the weakening of microbial resistance may result in increased resistance and poor bactericidal effects. The coating and photocatalytic anti-corrosion technology are useful in a way, but long-term effectiveness and safety are the key considerations. The coating method has fewer toxic materials and good environmental protection. Although some coating surfaces have achieved a certain hydrophobic effect, which can reduce the attachment of microorganisms, their construction and maintenance are quite difficult. A coating surface must be regularly cleaned underwater, otherwise it will increase drag and fuel consumption, and frequent underwater surface cleaning will cause damage to the coating surface and will increase roughness.

Therefore, in a pipeline system, some pipelines that are easy to maintain can be protected by physical and coating methods, which are low-cost and easy to construct. For some places that are difficult to maintain, especially for buried pipelines, the best anti-corrosion method is to combine photocatalysis and protective coating technology to produce a synergistic effect against microbial attachment, thereby providing a double-layer protection against bacterial attachment.

## 7. Summary and Outlooks

The study of the microbiologically influenced corrosion and protection of pipelines is an important area of research. This review presents the different types of corrosion and their consequences. In pipeline materials such as copper and steel, the main corrosion microorganisms are NRB and SRB, and the main cause of corrosion is pitting corrosion caused by ET–MIC or electrochemical corrosion. The major protective method for pipeline materials requires restricting biofilm formation on their surface. In addition, coating or antibacterial materials such as light and electrocatalysis are often adopted to prevent biocorrosion. In the case of non-metallic pipes, such as those made of PP and PV, the main corrosion microorganisms are some filamentous fungi or bacteria. Preventing the adverse effect of biocorrosion in non-metallic pipes requires using antibacterial agents in the preparation process. Presently, the research on the mechanism and influencing factors of pipeline microbial corrosion is still not clear and comprehensive, which also brings some problems to the research and development of protection technology. The following outlook can be drawn from this study:

(1) The diversity of microbial populations and the difference in environment within pipeline systems need further research. This review focuses on the mechanism of microbial corrosion in metal pipelines. Considering the increasing demand for pipelines in the construction industry, it is also necessary to summarize the corrosion problems caused by microorganisms in past pipeline systems. This will lay a theoretical foundation for research on the anti-corrosion of pipelines.
(2) Industrial environments, such as drinking-water factories, food-processing facilities, medical devices, oil transportation and drainage pipelines, and other environments, could produce heavy metals, leading to poor water-quality safety and harming human health. It should be noted that anti-corrosion technology research requires saving energy and protecting the environment.
(3) The development of survey technology is not detailed, and various biochemical parameters in the internal environment of pipelines are difficult to detect in a timely and accurate way. There is also no uniform standard for measuring large amounts of data obtained under specific experimental conditions. Thus, further policies and detailed information are required in this regard.

## Figures and Tables

**Table 1:** Common pipe materials and their applications, advantages, and disadvantages.

S/N	Material	Application	Advantages	Disadvantages
1.	Copper [30,36,37,38,39]	Air conditioning, plumbing, ships, medical field, etc.	Strong, stable, antimicrobial, and corrosion resistant	Expensive, poor processability, and low thermal conductivity
2.	Concrete [40,41,42]	Drain pipes and sewage	Cheap, high processability, and durable	Large weight and low corrosion resistance
3.	Carbon steel [43,44,45,46]	Water supplies, heating, and firefighting	Low cost and good mechanical properties	More susceptible to corrosion than stainless steel
4.	PE [47,48,49]	Water supplies	Chlorine resistant, smooth inner surface, lightweight, and higher corrosion resistance than PP pipes	Higher price than cast iron pipes and steel pipes, low heat resistance, and poor connection reliability
5.	PP [33,50,51]	Indoor warm and cooling water systems	Inexpensive, stable, and heat and corrosion resistant	Poor processability and high blockage rate
6.	Galvanized pipe tubes [52,53,54,55]	Fire pipes and pressure wastewater	Diverse and highly adaptable	Low corrosion resistance and easy to pollute water quality
7.	Cast iron [49,56,57]	Drain pipes and dewatering	Cheap, good ductility, and high corrosion resistance	Rusty, large weight, and poor processability and durability
8.	Stainless steel [33,36,50]	Sanitation facilities, drinking-water pipes, and air conditioners	Very strong, good thermal conductivity, durable, high corrosion resistance, and eco-friendly	Expensive and high rate of pitting corrosion
9.	PVC [54,58,59,60]	Wastewater and sewage	Good compressive characteristics, lightweight, processable, cheap, and high corrosion resistance	Poor heat resistance, low rigidity, and poor quality

## Data Availability

The original contributions presented in this study are included in the article, and further inquiries can be directed to the corresponding authors.
